# Downregulation of Transketolase Activity Is Related to Inhibition of Hippocampal Progenitor Cell Proliferation Induced by Thiamine Deficiency

**DOI:** 10.1155/2014/572915

**Published:** 2014-06-16

**Authors:** Yanling Zhao, Yiying Wu, Haolu Hu, Jinghui Cai, Min Ning, Xiushi Ni, Chunjiu Zhong

**Affiliations:** ^1^Department of Geriatrics, Shanghai First People's Hospital Affiliated Shanghai Jiaotong University, 100 Haining Road, Shanghai 200080, China; ^2^Department of Neurology, Zhongshan Hospital Affiliated Fudan University, 180 Fenglin Road, Shanghai 200032, China

## Abstract

In animal experiments, hippocampal neurogenesis and the activity of thiamine-dependent transketolase decrease markedly under conditions of thiamine deficiency. To further investigate the effect of thiamine deficiency on the proliferation of hippocampal progenitor cells (HPCs) and the potential mechanisms involved in this effect, we cultured HPCs in vitro in the absence of thiamine and found that proliferation and transketolase activity were both significantly repressed. Furthermore, specific inhibition of transketolase activity by oxythiamine strongly inhibited HPC proliferation in a dose-dependent manner. However, thiamine deficiency itself inhibited the proliferation to a greater degree than did oxythiamine. Taken together, our results suggest that modulation of transketolase activity might be one of the mechanisms by which thiamine regulates the proliferation of hippocampal progenitor cells.

## 1. Introduction

In recent years, an increasing number of studies support the view that neurogenesis in the mammalian central nervous system is an important component of learning and memory. The subventricular zone (SVZ) of the lateral ventricles and the subgranular zone (SGZ) of the hippocampal dentate gyrus have been established as the principal loci for potential neurogenesis in the central nervous system [1, 2]. In the hippocampus, which has long been considered the most important structure associated with cognition, it has been proven that neural progenitor cells located in the SGZ can persistently proliferate and mature into new adult neurons that integrate into neural circuits, where they participate in learning and memory. This is considered the cellular basis of cognition [3]. Thus, interference with neurogenesis in the hippocampus could result in cognitive deficits [4]. In general, many in vitro and in vivo factors such as neurotransmitter release, nutrition, environment, exercise, decrepitude, alcohol consumption, and radiotherapy [4–10] could affect hippocampal neurogenesis. Of these, nutrition has been the best studied.

Thiamine is a water-soluble vitamin that human beings can obtain only from exogenous sources such as grain and meat [11]. Thiamine deficiency (TD) with or without alcohol abuse can lead to Wernicke encephalopathy (WE), which is clinically characterized by progressive obstructive anterograde or retrograde amnesia and is the third most common condition that causes decline in cognitive function, after Alzheimer's disease and vascular dementia [12]. Even in Alzheimer's disease, thiamine levels are much lower than normal in 46% of patients in the early stages of the disease [13]. After absorption, thiamine is converted to thiamine pyrophosphate, which is the main active form of thiamine in vivo and functions as a coenzyme for the *α*-ketoglutarate dehydrogenase complex (*α*-KGDH), pyruvate dehydrogenase (PDH), and transketolase (TK) [14]. *α*-KGDH and PDH are two key mitochondrial enzymes involved in glucose metabolism and ATP generation by the Krebs cycle [15]. TK is the key enzyme in the pentose phosphate pathway (PPP) that synthesizes ribose-5-phosphate and the reduced form of nicotinamide adenine dinucleotide phosphate (NADPH) [12, 16]. Thiamine is essential for maintaining the Krebs cycle and the pentose phosphate pathway.

The levels of thiamine in the human brain are far lower than those in other organs or tissues, suggesting that the brain is highly sensitive to TD [12]. In our previous study, we found that hippocampal neurogenesis is significantly decreased in a TD mouse model established by feeding mice with a thiamine-depleted diet. Notably, hippocampal neurogenesis was greatly impaired and contributed to the cognitive dysfunction induced by TD at the early prepathological lesion stage [17]. TK activity and NADPH levels decreased more significantly in the hippocampus than in the cortex [18]. Considering that cell proliferation requires a large amount of ribose-5-phosphate for biosynthesis of nucleotides [19], a decrease of TK activity that impairs the PPP would decrease the metabolism of ribose-5-phosphate and NADPH, thereby inhibiting hippocampal neurogenesis.

Based on previously reported results, it was hypothesized that thiamine might regulate the proliferation of hippocampal progenitor cells (HPCs) by regulating the PPP. Previous studies suggest that oxythiamine, a TK inhibitor, can repress nucleic acid synthesis by downregulating the activity of the PPP [20]. In this report, neural stem cell media without thiamine or with added oxythiamine were used to investigate the effect of TD and inhibition of TK activity on the proliferation of HPCs, in order to explore the impact of TD on hippocampal neurogenesis and its possible mechanisms.

## 2. Materials and Methods

### 2.1. Ethics of Experimentation

All procedures performed on rats were in accordance with the Council of the European Communities Directive (86/609/EEC) for the care and use of laboratory animals.

### 2.2. Animals and Reagents

Sprague-Dawley rats at embryonic day 16 (E16) were obtained from the Shanghai Research Center for Experimental Animals, Chinese Academy of Science. All experiments were carried out according to the guidelines of the Animal Care Committee of Fudan University. All reagents were purchased from Sigma (St. Louis, MO, USA), unless otherwise noted.

### 2.3. Culture and Treatment of Hippocampal Progenitor Cells

Hippocampal progenitor cells (HPCs) were isolated and propagated using a neurosphere method as previously described [21, 22], with minor modifications. In brief, E16 rat embryos were isolated from their mothers under deep anesthesia and placed in ice-cold sterile D-Hank's balanced salt solution. Hippocampal tissues were carefully microdissected using a stereomicroscope and triturated with a Pasteur pipet to obtain a single-cell suspension. The cells were then centrifuged at 1000 g for 5 min. The supernatant was removed, and the viable dissociated cells were seeded into uncoated 100 mm dishes at a density of 5 × 10^4^ cells/cm^2^. The medium contained a mixture of Dulbecco's modified Eagle's medium and F-12 nutrient (1 : 1, v/v), supplemented with 20 ng/mL epidermal growth factor, 20 ng/mL fibroblast growth factor-2, and 2% B-27. The thiamine concentration in the medium was 6.4 *μ*M/L. HPCs were maintained in a humidified incubator at 37°C under an atmosphere of 95% air/5% CO_2_, and 50% medium changes were carried out every 2-3 d. Neurospheres were passaged by trituration every 6-7 d.

After a minimum of three passages, single-cell HPC suspensions were cultured in thiamine-deficient medium (TD group) and in normal medium, that is, with 6.4 *μ*M/L thiamine (control group). Simultaneously, single-cell suspensions were cultured in normal medium containing various concentrations of oxythiamine (0, 0.5, 5, 10, and 50 *μ*M/L).

### 2.4. Number and Size of Neurospheres

Single-cell HPC suspensions were seeded into uncoated 96-well plates at a cell density of 2000 cells per well and allowed to grow for 7 days. Neurospheres were imaged at 50x magnification. Cell numbers and diameters were measured using Image Pro Plus 6.0.

### 2.5. CCK-8 Assay

Indirect counting of viable cells was carried out by CCK-8 assay using a Cell Counting Kit-8 (Dojindo, Rockville, MD, USA). Briefly, single-cell suspensions were prepared from neurospheres and plated into uncoated 96-well plates at a cell density of 5000 cells per well. After 4 and 7 days of incubation, 10 *μ*L of the Cell Counting Kit solution was added to each well and left to incubate for an additional 5 h at 37°C. The absorbance at 450 nm was then measured and the results were expressed as optical density (OD).

### 2.6. Bromodeoxyuridine- (BrdU-) Incorporation Assay

Single-cell suspensions were plated on coverslips coated with poly-L-ornithine (20 mg/mL) and laminin (5 mg/mL) at a cell density of 5000 cells/coverslip, allowed to grow for 4 days, and then labeled with 10 *μ*M/L BrdU for 15 h. The cells on the coverslips were then fixed in 4% paraformaldehyde for 30 min and placed in 0.1 M PBS, pH 7.4. Blocking was carried out using 5% normal horse serum with 0.5% Triton X-100 for 1 h. The cells were then incubated with rat anti-BrdU antibody (1 : 500, Abcam, Cambridge) at 4°C overnight. After washing, the cells were incubated with fluorescein isothiocyanate (FITC) rabbit anti-rat antibody (1 : 500) for 1 h at room temperature. Nuclei were stained with Hoechst 33342 for 5 min. Immunostained samples were visualized under a Leica DMIRB microscope. BrdU-positive cells were counted in a visual field (about 1 mm^2^) at 100x magnification, and the results were expressed as a percentage of the total cells (determined by Hoechst 33342 staining).

### 2.7. TK Activity

After 4 days of incubation, cells were homogenized with ice-cold 0.1 M Tris-HC1 buffer (pH 7.6) and centrifuged at 16**200 g for 15 min, and the supernatant was collected. The protein concentration was determined using the BCA Protein Assay Kit and recorded as C (g/L).

TK activity was measured as described by Bayoumi and Rosalki, with small modifications [23]. Enzyme activity was measured by adding 50 *μ*L of supernatant to 200 *μ*L of the reaction mixture containing 14.4 mM/L ribose-5-phosphate, 190 *μ*M/L NADH, 380 *μ*M/L TPP, >250 U/L glycerol-3-phosphate dehydrogenase (GDH), and >6500 U/L triose phosphate isomerase. The optical density (OD) was measured at 340 nm immediately and then once every 5 min thereafter for 1 h. The activity was deduced from the difference between the absorbances measured at 15 and 45 min. One unit of enzyme activity is defined as the amount of enzyme that catalyzes the oxidation of 1 *μ*mol of NADH per min. The enzyme activity assay was repeated three times for each group and an average value was obtained. The TK activities (%) of the treated group were normalized to those of the control group (100%).

### 2.8. Statistical Analysis

Results were expressed as mean ± SEM. Differences between the TD and control groups or between various concentrations of oxythiamine were analyzed using one-way analysis of variance and considered statistically significant when *P* < 0.05.

## 3. Results

### 3.1. Effect of Thiamine Deficiency on the Proliferation of HPCs

**Figure 1:**
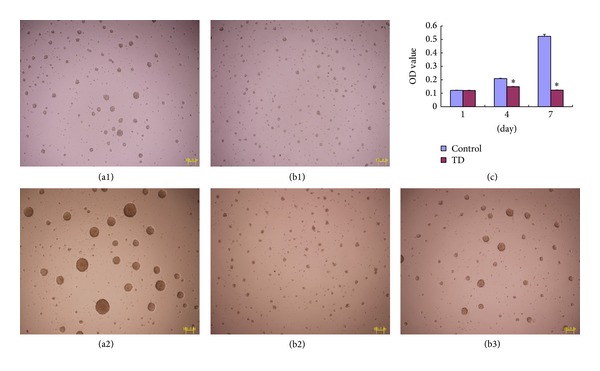
Effect of thiamine deficiency on the proliferation of hippocampal progenitor cells (HPCs). Neurospheres of the thiamine deficiency (TD) group ((b1), 4 d of culture; (b2), 7 d of culture) were much smaller than those of the control group ((a1), 4 d of culture; (a2), 7 d of culture). On the seventh day of culture, the structure of the neurospheres of the TD group became porous, but their size increased and their structure became tight after replenishment of thiamine (b3). The optical density (OD) value of the TD group in the CCK-8 assay, indicating viability, was significantly lower than that of the control group by the fourth day (c). Scale = 100 *μ*m.

Our morphological analysis showed that on the fourth day of culture, the neurospheres in the TD group (Figure 1(b1)) were obviously smaller than those in the control group (Figure 1(a1)). On the seventh day, the neurospheres in the control group showed a continuous increase in size during incubation (Figure 1(a2)), whereas the neurospheres of the TD group had become smaller and more porous (Figure 1(b2)). When thiamine was replenished, the neurospheres of the TD group began increasing in size again as revealed at a time point 3 days after replenishment (Figure 1(b3)).

Results obtained by CCK-8 assay show that the proliferation of HPCs significantly decreased under TD (Figure 1(c)). On the first day of culture, the OD values showed no significant difference between the TD group and the control group (0.1215 ± 0.0015 versus 0.1204 ± 0.0019, *P* > 0.05, *n* = 6). On the fourth day of culture, the OD value of the TD group was significantly lower than that of the control group (0.1489 ± 0.0019 versus 0.2091 ± 0.0020, *P* < 0.05, *n* = 6). On the seventh day of culture, the difference between the OD values of the TD group and the control group was more significant (0.1233 ± 0.0012 versus 0.5231 ± 0.0144, *P* < 0.05, *n* = 6). In contrast with the continuous increase in the OD value observed in the control group, no significant change was seen in the TD group, demonstrating that the proliferation of the HPCs had been totally inhibited. However, the OD value of the TD group increased to 0.2787 ± 0.0051 three days after replenishment of thiamine.

**Figure 2:**
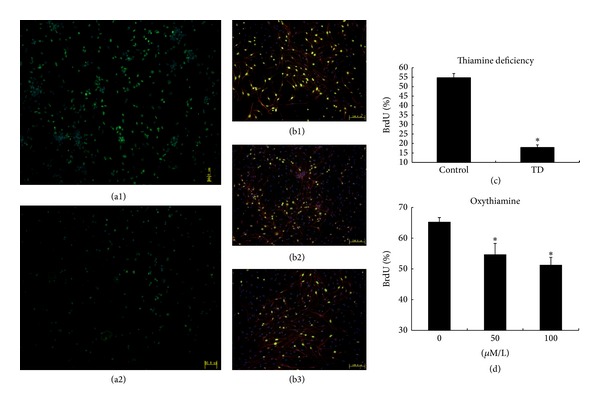
BrdU incorporation. Green represents BrdU-positive cells, red represents cells positive for nestin (a specific marker of HPCs), and blue represents nuclei stained with Hoechst. (c) Analysis of the TD group; the proportion of BrdU-positive cells of TD (a2) was significantly lower than that of the control group (a1). (d) Analysis of the oxythiamine-added groups; the proportion of BrdU-positive cells in HPCs treated with 50 *μ*M/L (b2) and 100 *μ*M/L (b3) oxythiamine was significantly lower than that of the 0 *μ*M/L group (b1). (a1) and (a2): scale = 50 *μ*m; (b1), (b2), and (b3): scale = 100 *μ*m.

The results on BrdU incorporation similarly showed that the proportion of BrdU-positive cells in the TD group on the fourth day of culture was significantly lower than that of the control group (17.9382 ± 1.3317% versus 54.74339 ± 2.1774%, *P* < 0.05, *n* = 6; Figures 2(a2), 2(a1), and 2(c)).

### 3.2. Effect of Oxythiamine on HPC Proliferation

**Figure 3:**
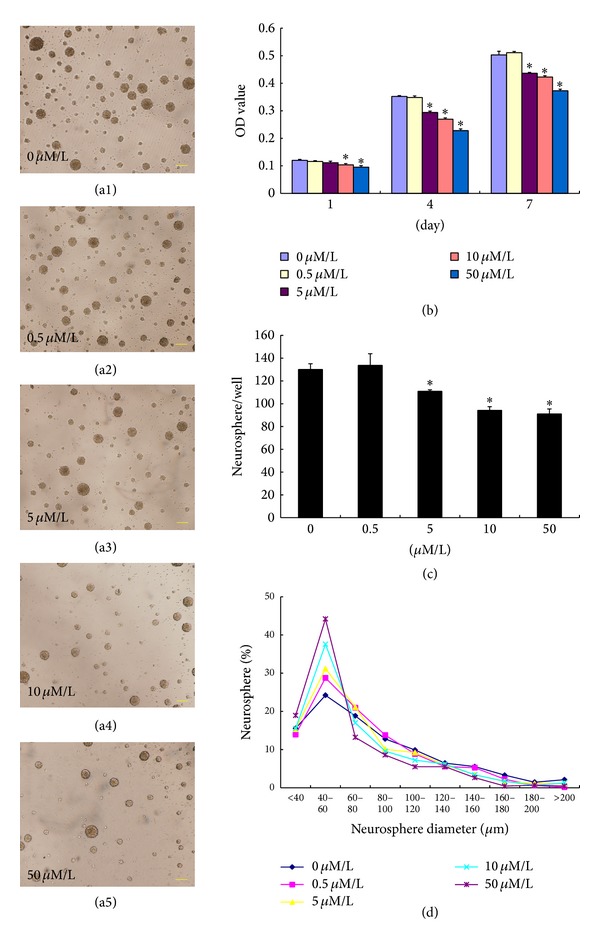
Inhibition of the proliferation of HPCs by oxythiamine. The number of neurospheres significantly decreased in a dose-dependent manner in HPCs treated with >5 *μ*M/L oxythiamine ((a1)–(a5), (c)), and the proportion of small neurospheres was in direct proportion to the oxythiamine dose (d). The OD values generated by the CCK-8 assay of HPCs treated with oxythiamine at doses >5 *μ*M/L were significantly lower than those of the 0 *μ*M/L group (b). Scale = 100 *μ*m.

To investigate whether thiamine regulated the proliferation of HPCs by inhibiting the activity of TK, oxythiamine was applied to HPCs during cultivation in vitro. Comparing the neurospheres of different groups with different doses of oxythiamine, we found that neurospheres of all groups enlarged over time. However, the growth rate of the neurospheres varied according to the concentration of oxythiamine. On the seventh day, the number of neurospheres began to gradually decrease with increasing oxythiamine dose (5–50 *μ*M/L) compared to the control group (0 *μ*M/L, *P* < 0.05, *n* = 6; Figures 3(a1)–3(a5) and 3(c)). Moreover, the number of small neurospheres was in direct proportion to the concentration of oxythiamine (Figure 3(d)).

Results obtained by CCK-8 analysis (Figure 3(b)) showed that the OD values of HPCs treated with different doses of oxythiamine were 0.1200 ± 0.0040 (0 *μ*M/L), 0.1159 ± 0.0030 (0.5 *μ*M/L), 0.1115 ± 0.0060 (5 *μ*M/L), 0.1038 ± 0.0040 (10 *μ*M/L), and 0.0954 ± 0.0060 (50 *μ*M/L) on the first day of culture, respectively. The OD values of HPCs treated with 10 *μ*M/L and 50 *μ*M/L oxythiamine were significantly lower than those of the control group (0 *μ*M/L; *P* < 0.05, *n* = 6). On the fourth day, the OD values of the different groups were 0.3523 ± 0.0030 (0 *μ*M/L), 0.3483 ± 0.0059 (0.5 *μ*M/L), 0.2938 ± 0.0050 (5 *μ*M/L), 0.2693 ± 0.0050 (10 *μ*M/L), and 0.2279 ± 0.0060 (50 *μ*M/L), indicating a negative correlation between oxythiamine dose and HPC proliferation, and HPCs treated with >5 *μ*M/L oxythiamine showed a more significant inhibition of proliferation (*P* < 0.05, *n* = 6). On the seventh day of culture, the OD values of the different groups were as follows: 0.5033 ± 0.013 (0 *μ*M/L), 0.5113 ± 0.0050 (0.5 *μ*M/L), 0.4365 ± 0.0030 (5 *μ*M/L), 0.4225 ± 0.0050 (10 *μ*M/L), and 0.3727 ± 0.0060 (50 *μ*M/L) respectively, and HPCs treated with >5 *μ*M/L oxythiamine showed significantly lower proliferation rates (*P* < 0.05, *n* = 6).

In a BrdU-incorporation assay that was run on the fourth day of culture, HPCs treated with 50 *μ*M/L oxythiamine (54.7 ± 3.58%) and 100 *μ*M/L oxythiamine (51.3 ± 2.46%) showed significantly decreased proportions of BrdU-positive cells compared to those of the control group (65.31 ± 1.4%, *P* < 0.05, *n* = 6; Figures 2(b2), 2(b3), 2(b1), and 2(d)).

### 3.3. Analysis of TK Activity

**Figure 4:**
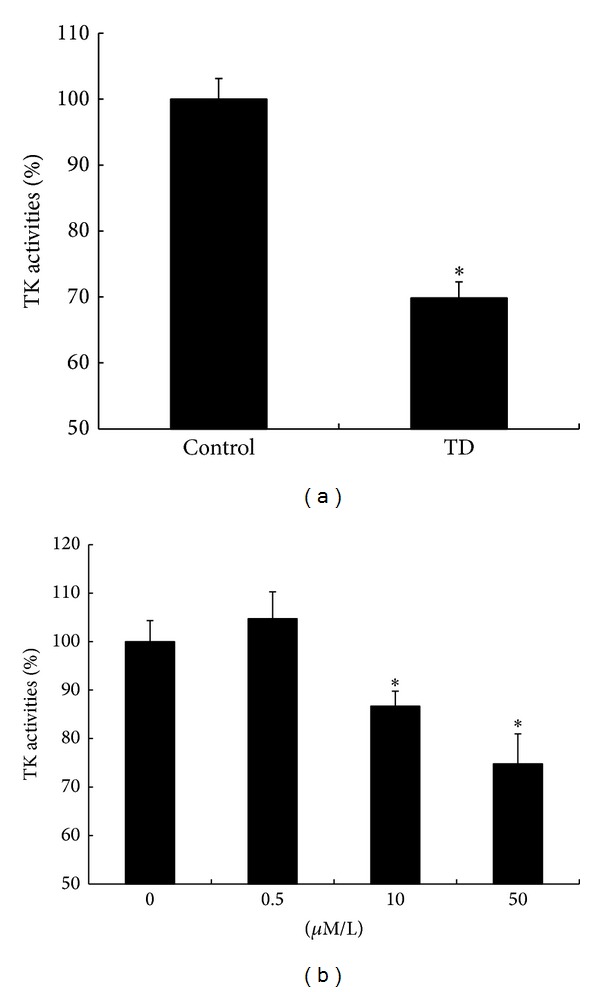
Analysis of TK activity. The TK activity of the TD group was significantly lower than that of the control group (a). There was no significant difference between the TK activity of HPCs treated with 0.5 *μ*M/L oxythiamine and that of the 0 *μ*M/L control group; however, the TK activity decreased with increases in oxythiamine dose (≥10 *μ*M/L; (b)).

On the fourth day of culture, the TK activity of the TD group was significantly lower than that of the control group (69.86 ± 2.43% versus 100 ± 3.14%, *P* < 0.05, *n* = 5; Figure 4(a)). The TK activity of the HPCs treated with 10 *μ*M/L and 50 *μ*M/L oxythiamine (86.66 ± 3.14% and 74.78 ± 6.20%, resp.) was significantly decreased relative to that of the control group (100 ± 4.31%, 0 *μ*M/L), but that of the HPCs treated with 0.5 *μ*M/L oxythiamine (104.71 ± 5.54%) did not show significant change (Figure 4(b)).

## 4. Discussion

In this study, we cultured HPCs in neural stem cell medium without thiamine and found, using the CCK-8 and BrdU-incorporation assays, that the proliferation of HPCs was significantly inhibited under these conditions. During 7 days of culture without thiamine, the proliferation of HPCs was almost completely inhibited, in contrast to the control group (shown in Figure 1(c)), and the resulting neurospheres were smaller and more porous. The proliferation capacity of the treated HPCs could subsequently be rapidly restored by replenishing the thiamine. Taken together, these results suggest that thiamine is of great importance to the proliferation of HPCs, and this finding is consistent with our previous report that neurogenesis was significantly impaired in the hippocampus of a TD mouse model [17]. Furthermore, a similar phenomenon was observed in an animal model of WE treated with pyrithiamine, an antagonist of thiamine [24]. Numerous reports have demonstrated that hippocampal neurogenesis is heavily implicated in hippocampus-mediated cognitive function [25, 26]. The neural stem cells proliferate and differentiate, especially into cholinergic neurons, to form the cellular basis of cognition. In hippocampus and cortex of TD animal models, loss of cholinergic neurons and alterations in stimulated acetylcholine (ACh) levels were also noticed and were considered contributing factors for cognitive impairment [27–29]. Moreover, TD induces a loss of regulatory cholinergic input to the hippocampus [24]; conversely, physiological or pharmacological cholinergic stimulation may support adult hippocampal neurogenesis [30]. An impaired cholinergic system might contribute to the decrease of hippocampal neurogenesis caused by TD. In the present report, we first tested the negative effect of TD on cultured HPCs in vitro. Because of inhibition of TK activity in TD condition, the proliferation of HPCs in vitro was inhibited greatly and the differentiation of HPCs into neurons, especially into cholinergic neurons, may be impaired, although elucidation of the details will require further study.

As a coenzyme of KGDH, PDH, and TK, thiamine can regulate the proliferation of HPCs by regulating the activity of these enzymes. The findings of our previous animal experiments suggested that TD led to significant reduction in the activity of TK, but not PDH or KGDH, in the hippocampus [18]. TD resulting from chronic alcohol abuse is one factor underlying alcohol-related brain damage such as WE. Bouts of TD may occur in upwards of 80% of patients with alcoholism. However, only about 13% of such individuals develop WE, raising the possibility that a genetic predisposition to WE may exist in some individuals [31]. Meanwhile, some studies have shown that TK binds TPP less effectively in patients with WE than in healthy controls [32]. These data imply that TK may play an important role in the brain damage following TD. TK is the key enzyme in the PPP for the synthesis of NADPH and ribose-5-phosphate and for maintaining the redox state of glutathione. In a mouse model of diabetes, TK activity in the heart was found to be greatly decreased [33], and the proliferation of heart stem cells was significantly inhibited [34]. After supplementation with benfotiamine, a derivative of thiamine, TK activity resumed in this model and the proliferation of heart stem cells was restored because of reactivation of the PPP and restoration of the redox state of heart stem cells [35]. These observations suggest that TK and the related PPP play an important role in regulating the proliferation of nondifferentiated cells. In order to evaluate whether TK is involved in the regulation of hippocampal neurogenesis in TD, HPCs were treated with oxythiamine, a TK inhibitor. Compared to the control, the number and size of the neurospheres were greatly decreased. These results were supported by the results of the CCK-8 and BrdU-incorporation assays, showing that oxythiamine repressed the proliferation of HPCs in a dose-dependent manner. Furthermore, TK activity was greatly inhibited, implying that TK and the PPP were the key factors for thiamine regulation of the proliferation of HPCs.

In addition to the inhibition of TK activity, both TD and oxythiamine had a negative effect on the proliferation of HPCs. On the fourth day of culture, the decrease of TK activity in the HPCs receiving TD treatment was roughly identical with the effect of culture with 50 *μ*M/L oxythiamine; however, the proliferation of HPCs under the former condition was more severely inhibited than under the latter. This was also demonstrated by morphological analysis. On the fourth day of culture without thiamine, the size of the neurospheres in the TD group was much smaller than that in the control group, and, by the seventh day of culture without thiamine, the size of the neurospheres in the TD group had still not increased and their structure had become spongiform. In contrast, there was no obvious difference in appearance between experimental and control groups by day four in HPCs exposed to 50 *μ*M/L of oxythiamine, and, by the seventh day, though the neurospheres had greatly decreased in number and size, their structure remained compact. The possible reasons for this might be as follows: (1) compared to HPCs treated with TD, only the TK activity was inhibited in the oxythiamine-added groups and (2) even if the TK activity had been comparably inhibited by oxythiamine and TD, the proliferation pattern of the HPCs was different under the two conditions, implying that other mechanisms were involved. Although the TD condition can inhibit other enzymes, including the two key mitochondrial enzymes PDH and KGDH, thiamine can reportedly abrogate hippocampal neurogenesis inhibition by mitochondrial inhibitors [36], suggesting that thiamine deficiency might negatively regulate hippocampal neurogenesis by altering mitochondrial function in parallel with its effect on TK.

According to epidemiological surveys, the elderly are more prone to TD, and the incidence of TD in older people is approximately 23–40% in hospitals [37, 38]. Moreover, thiamine levels decreased with age and were much lower in the elderly than in the young [39]. While senescence might be the major reason for cognitive hypofunction, lower levels of thiamine in the elderly might be another important reason for this [40]. It has been reported that 13.3% of older people with cognitive dysfunction resulting in acute behavior disorders suffered from thiamine deficiency [41]. An increasing number of studies confirm that decreased hippocampal neurogenesis is accompanied by senescence and is closely correlated with cognitive dysfunction [42, 43]. Moreover, the transplantation of neural stem cells and other exogenous interventions aimed at improving hippocampal neurogenesis has been found to significantly improve learning and memory in the senescent mouse [44, 45]. Based on the results of this report, thiamine administration might be another candidate therapy for enhancing hippocampal neurogenesis and improving the cognitive functions of older individuals; however, this requires further investigation.

In this study, we investigated the proliferation of HPCs under conditions of TD or oxythiamine exposure. We proved for the first time that oxythiamine, an inhibitor of TK, significantly inhibited the activity of TK specifically in HPCs and also inhibited their proliferation, although the effect of the inhibitor on proliferation was less prominent than that of actual TD. The results suggest that TK might be a key enzyme in the inhibition of the proliferation of HPCs induced by TD—a finding with considerable pharmacological potential.
